# From integrated to fragmented elites. The core of Swiss elite networks 1910–2015

**DOI:** 10.1111/1468-4446.12929

**Published:** 2022-02-14

**Authors:** Thierry Rossier, Christoph Houman Ellersgaard, Anton Grau Larsen, Jacob Aagaard Lunding

**Affiliations:** ^1^ Department of Sociology London School of Economics London UK; ^2^ Department of Organization Copenhagen Business School Frederiksberg Denmark; ^3^ Faculty of Social and Political Sciences, Institute of the Social Sciences University of Lausanne Lausanne Switzerland; ^4^ Department of Social Sciences and Business Roskilde University Roskilde Denmark

**Keywords:** coordination, elites, historical sociology, inequality, networks, social networks

## Abstract

This article focuses on historical elite dynamics and investigates elites' integration over time. We describe the changing relations and composition of the central circles in Swiss elite networks at seven benchmark years between 1910 and 2015 by relying on 22,262 elite individuals tied to 2587 organizations among eight key sectors, and identify for each year the most connected core of individuals. We explore network cohesion and sectoral bridging of the elite core and find that it moved from being a *unitary corporate elite*, before 1945, to an *integrated corporatist elite*, between the 1950s and 1980s, before *fragmenting into a loose group*, with an increased importance of corporate elites, in the 1990s onwards. The core was always dominated by business and their forms of legitimacy but, at times of crisis to the hegemony of corporate elites, after the Second World War and (only) shortly after the 2008 financial crisis, elite circles expanded and included individuals with delegated forms of power, such as politicians and unionists. In the most recent cohort (2015), the share of corporate elites in the core is similar to the one before the First World War and during the interwar period. This *return to the past* echoes findings on wealth inequality and economic capital accumulation by a small group of individuals organized around the most powerful companies.

## 1 INTRODUCTION

How do elites change and why? While we can no longer claim that elites are “forgotten by the social sciences” (Savage & Williams, 2008)**,** the resurgence of the sociology of elites still leaves many paths to be explored (Cousin et al., 2018). In particular, the temporal dynamic of elites remains open for inquiry. In light of how focusing on inequality during a long period of time has enabled economists such as Piketty (2014, 2020; Paidipaty & Savage, 2021; Savage & Waitkus, 2021) to reinvigorate debates on the links between capital accumulation and wealth inequality, this paper aims to take up Piketty’s challenge to sociology (Savage, 2014) and describe elite dynamics. This study is concerned about elite groups coordinating through vast organizational networks and across different sectors over time. For the first time, we can analyze, over a historical period of more than a century, how relations between elite groups evolve and how the composition of cross‐sectorial elites defined dynamically through elite networks change. We are able to show that the business elites running the companies linked to thorough processes of wealth accumulation have the primacy over other elite groups during the whole 20th century. More importantly, we show that while the power of this group has been mitigated by the prominence of other (political, administrative, union) elites after the Second World War, through the intensification of neo‐corporatist processes fostered by the state, and shortly (only) after the 2008 financial crisis, the increased influence of the corporate elites at the end of the 20th and beginning of the 21st century reminds us of the pre‐war era and the related imperial forms of accumulation. In that sense, and in line with Savage’s (2021) work on the historical importance of wealth inequality and economic capital accumulation by a small group of individuals organized around the most powerful companies, we observe a *return to the past*, where the corporate fraction of the elites are, again, as much prominent now as they were more than a century ago.

We explore the changing relations and composition of the central circles in Swiss elite networks by relying on a total of 22,262 elite individuals tied to 2587 elite organizations from eight key sectors (business, unions, politics, public administration, expert committees, academia, other organizations and associations and the military) divided into seven historical cohorts (1910, 1937, 1957, 1980, 2000, 2010 and 2015). By looking at the interlocks between elite groups and identifying those who integrate most frequently, we describe how the dynamics of the central social circle in Switzerland evolved during the 105 years studied. We explore the level of integration and the degree of cross‐sectoral bridging in elite circles, showing how the Swiss elite networks’ core (778 individuals in total) moves from being dominated by a *unitary corporate elite* before World War 2 to an *integrated elite* bridging several sectors until the 1980s, before the *network core fragmented*, with a renewed importance of corporate elites. The core was always dominated by business, but the level of integration and strength of business *vis‐à‐vis* other groups differed as challenges to elites changed. At times of crisis to the hegemony of corporate elites, after World War II and right after the 2008 financial crisis, elite circles expanded and became more diverse, including elites with delegated forms of power, such as politicians and unionists. The Swiss case is relevant, since it is a strongly decentralized system and a particularly internationalized economy. In this context, one could expect the elites to be only marginally integrated, but our findings on elite cohesiveness challenge this assumption.

In the next part, we contextualize the evolution of Swiss elites, review the literature on historical elite studies and discuss the conceptual dimensions of elite integration and bridging, before introducing how we built the network and our key indicators. Then, we present our findings on the historical integration and sectoral bridging dynamics of the core. Finally, we summarize our results and discuss the relevance of the Swiss case regarding the most recent findings on wealth and social inequalities.

## 2 BACKGROUND: STUDYING ELITES OVER TIME

### 2.1 Transformations of Swiss elites

We introduce a periodization of the changing elite relations in Swiss society. Several particularities of Switzerland make it an interesting case for elite scholars. Within this strongly decentralized and polycentric context, with a weak central state and a particularly internationalized economy (Kriesi, 1998), one could expect elites to be only marginally integrated, but findings on elite cohesiveness challenge this assumption. A small elite group, often linked to business, concentrates a large share of power. Weak trade unions compete with organized export‐oriented employers supported by influential business associations (Katzenstein, 1985). Swiss elites share a set of common characteristics and do not integrate easily new groups into the power structure (Ginalski, 2016). Based on extant literature, three periods have been identified in the recent history of Switzerland corresponding to three distinct features of elite coordination and networks during the 20th and 21st centuries: (1) A *consolidation* period of elite relations, from the early 20th century until World War 2; (2) An *integration* period, from World War 2 until the 1980s; (3) A *fragmentation* period, from the 1990s onwards.

Since the end of the 19th century and until 1945, relations between Swiss elites followed a *consolidation* logic. The small size of the country led to the formation of a small elite group, who knew each other through organizational networks, leading to compromises between the main interest groups, political parties and the administration (Kriesi, 1980). Top company owners started to organize collectively to defend their class interests. They built dense corporate networks around financial firms that funded industrial companies and were supported by business associations (whose executive committee was formed of top company leaders and permanent secretaries) (Mach et al., 2016). Board directors developed strong organizational ties with other elite members (elected politicians, senior servants, renowned professors). Elite coordination intensified through meetings in various organizational structures, such as associations, party committees, the parliament (David et al., 2009; Eichenberger & Ginalski, 2017) or state expert committees (Bühlmann et al., 2017). Elites were strongly interrelated, often multipositionnal across various institutions, and even multisectorial (Bühlmann et al., 2012a), since *for example,* the lay parliamentary system allowed politicians to sit on other elite positions (Pilotti, 2017).

After World War 2, and until the 1980s, Swiss elites followed a sustained *integration* logic. The neo‐corporatist system of expert committees (“extra‐parliamentary commissions”) increased exponentially. These groups, majoritarily composed of non‐civil servants, are in charge of advising federal authorities, preparing legislation when implementing new laws or executing tasks for the state. In these committees, business elites met with politicians, university professors or other experts to coordinate on a particular topic (Rebmann & Mach, 2013). Given the growing importance of expert committees, elite coordination intensified across organizations and key sectors, and new groups were included. During the 1930s, unions (and their political ally the Swiss Socialist Party) and farmer organizations started to be included in neo‐corporatist processes and their involvement grew bigger after the war (Rebmann, 2011). Coordination and cohesiveness within and between elite groups culminated between the 1950s and the 1980s.

From the 1990s onwards, elite coordination experienced a *fragmentation* dynamic. Financialization led the close connections between bankers and industrialists to disappear and banks became less prominent in interlocks (David et al., 2015). The private sector experienced an internationalization process. New transnational managers had fewer incentives to be integrated into Swiss networks (Bühlmann et al., 2012b) and relied on new strategies to sidestep traditional political channels: e.g.*,* by founding in 1999 the US‐like think tank *Avenir Suisse*, which defended their interests through lobbying activity and the media (Mach et al., 2021). Swiss multinational companies lost centrality due to economic turmoil in the 2000s. The country’s major airlines company Swissair went bankrupt and was absorbed by the German Lufthansa. During the 2008 financial crisis, the biggest banks were hit severely (UBS had to be bailed out by the central bank). Since the 1990s, the parliament underwent a professionalization process and elected officials sat less in other organizations (Pilotti, 2017), while academia became more autonomous from political and economic powers, for example, for professors’ appointment (Horvath, 1996). Neo‐corporatist processes through expert committees declined in importance (Beetschen & Rebmann, 2016). We add to the description of Swiss elites by exploring how these changes and periodization are reflected in the structure and composition of the central circles in elite networks in terms of sectoral representation.

### 2.2 Historical elite studies

Processes of accumulation of capitals, resources, and assets by influential elites have been documented (Savage et al., 2005; Toft, 2019). As accumulation of different forms of capital often takes more than one generation, it is necessary to study elites historically (Nichols & Savage, 2017). This has even extended to historical analysis of changes in the upper classes (Scott, 1982) or relations between different power forms within large scale societies (Mann, 1986). Many elite studies have used historical sources to qualitatively describe changes in elite relations over time (Accominotti et al., 2018; Baltzell, 1958; Coleman, 1973; Mills, 1956). Through these studies, we understand how changes in elite relations reflect changes in the division of power across societies.

Changes in elite composition have been studied for long, from changes in the cabinet in the UK (Laski, 1928) and US (Mintz, 1975)—including links between cabinet and business (Freitag, 1975; Gill, 2018)—, or within business elites (Bendix & Howton, 1957; Friedman & Tedlow, 2003; Kaelble, 1980; Mills, 1945). Within the prosopography tradition (Broady, 2002; Lunding et al., 2020; Rossier, 2019 ), scholars studied collective biographies of elite groups spanning centuries, for example, on the Russian clergy (Plamper, 2000) or Finish business elites (Kansikas, 2015, 2016), focusing on changes in social background, education, career trajectory and, sometimes, gender. It is shown how elites have slowly opened up to newcomers, while remaining very selective. Most studies focus on elites defined positionally, but do not center on whether or not the set of positions included, their inclusion criteria, and internal relations evolved over time. Thus, if new groups enter the elites, these newcomers would not be included in these studies.

Studies analyzing evolution in relations within and between elite groups are less frequent, because of the difficulties of gathering relational data used for historical comparisons. Scholars have studied the evolution and recent decline of national corporate networks (Chu & Davis, 2016; Heemskerk & Fennema, 2009; Mizruchi, 1982; Mizruchi & Stearns, 1988). A conclusion is that, as political opposition to business interests from government or well‐organized unions declines, so does the internal cohesion of the corporate inner circle (Mizruchi, 2013; Useem, 1984). However, while national corporate networks seem to fragment, transnational corporate interlocks are on the rise (Carroll & Fennema, 2002; Heemskerk, 2013; Murray, 2017). Other scholars mapped changing field dynamics within business elites (Bühlmann et al., 2012b; François et al., 2016; Timans, 2015) and evolving relations between elite groups through cross‐sectoral flows of elite careers in France in 1969 and 2009 (Denord et al., 2018). Savage (2021) argues that social fields, where powerful field‐specific elites exerted their power, increasingly lost part of their autonomy during the last decades. Inheritors, especially those who own wealth and other forms of economic capital, are now able to convert their advantages to an “objectified” form that can be stored and passed on. This process results in the incursion of economic capital into other forms of (cultural, social, political and symbolic) capitals and in “entropy” mechanisms in all fields, while (economic) capital, accumulated in the global social space, gains in prevalence.

This study adds to this literature a focus on relations between elite groups, which maps changing dynamics in elite composition over more than a century. Our data do not have the granularity to focus on gradual year‐to‐year century long changes as recent studies of the 1% share of the income distribution (Alvaredo et al., 2013), class origins and educational achievements (Hansen & Strømme, 2021), elite schooling (Reeves et al., 2017) or elite cultural tastes (Friedman & Reeves, 2020), but allow us, for the first time, to analyze how relations *between* elite groups change, and how the composition of a cross‐sectoral elite defined dynamically through elite networks evolve. We add to the discussions on how changes in elite composition are related to political developments, not least the evolving relations between the capitalist class and state and union powers (Mizruchi, 2013).

## 3 THEORY: A TYPOLOGY OF ELITE NETWORK INTEGRATION

Elite integration is realized through their connections in vast organizational networks. Elites meet, collaborate and coordinate through affiliations to top institutions, which makes them become cohesive and bridge across sectors, depending on the importance and diversity of network ties (Domhoff, 1967; Windolf, 1998). The changing composition, as well as integration and sectoral bridging levels, at the core of elite networks allow us to explore elite changes. Identifying central actors and organizations in affiliation networks offer a blueprint of the power structure (Domhoff, in Denord et al., 2020). Mills' (1956) description of the power elite constitutes an empirical study on the particular balance of power in the 1950s in the US. He emphasizes how changes in the American power structure came about by institutional shifts in the relative positions—or different types of sectoral bridging—of the political, the economic, and the military orders (Mills, 1956). The three institutional orders—to which he would add kinship and religion in other societies (Denord & Réau, 2014; Gerth & Mills, 1953)—assumed historically dominant or subordinated roles within the power elite, in which the political order could also include organized farmers or unions. The power elite is constituted by “a set of overlapping circles” (Mills, 1956, p. 283) who manage to develop “coinciding interests”, and the question of who composes the power elite cannot be defined theoretically (Mills, 1956, p. 277), but must be explored empirically. This entails looking at the sectoral composition of the central circles in national elite networks and at this group’s level of embeddedness. Historically, US elites moved from pluralist “loose coalitions” in the early nineteenth century to “a heavy overlapping among the members of these several elites”—the integrated power elite—around 1950 (Mills, 1956, p. 269ff). In between these periods, more unitary and sectoral elites achieved supremacy, such as economic elites during the Gilded Age, and political elites during the New Deal period.

We distinguish between the network embeddedness (Granovetter, 1985) of elites and the degree elites are embedded across sectors to other elites. Elite networks' cohesiveness and the propensity of their ties to cut across sectors indicate the extent to which elites are involved in reciprocal commitments of loyalty to individuals both from their own field and in other sectors. Cohesive networks among corporate elites allow for the creation of a “classwide rationality” making key business leaders represent the capitalist class as a whole in other arenas of power (Useem, 1984), but these networks may fragment since their political success can render the ability to act in concert—which requires network embeddedness—obsolete (Mizruchi, 2013). Identifying central individuals and affiliations is one possible way to empirically describe which influential groups overlap and which role sectoral elites play in the power structure. By using elite networks to identify groups with the highest social overlap, we use what Mills (1956, p. 11) calls the “mutual attraction among those who ‘sit on the same terrace’” to identify central circles of elites as the network core within a larger elite group, following a key question: What is the level of integration in elite networks both within and between sectoral elites?

In Table 1, we present a typology of these two forms of elite integration within the core. *Fragmented elites* primarily act in the interest of their own organizations having few structural constraints (Mizruchi, 2013). *Loose‐knit elites* are weakly embedded with relatively strong ties to other sectors and are akin to pluralist elites differentiated by sector (Aron, 1950; Dahl, 1961), but united in search for compromise (Higley & Burton, 1989; Moore, 1979). *Unitary elites* are well‐integrated in their own sector and dominate the central circle of the network. They take on “inner circle”‐like qualities (Useem, 1984): they can express interests on behalf of the whole group and act in concert, and are only weakly structurally constrained by the interests of other elite groups. *Integrated elites* are both internally and cross‐sectorally embedded. They are committed to the interests of their own sector and have well‐established alliances with other key groups forming a power elite‐like coalition (Mills, 1956). Looking at the integration level within elite central circles entails identifying the core group in elite networks and analyzing their cohesiveness and capability to broker between sectors. The type of sectors included in the core allows us to assess which institutional orders dominate an elite constellation. If a fragmented or unitary elite is identified, one sector dominates the others, while the distribution of sectors in loose‐knit and integrated elites is indicative of their relative strength. In the less integrated elite types—fragmented and loose‐knit elites—, the mutual commitment of the sectors, organizations and individuals in the core is much weaker. This may be because an elite group has established such a strong hegemony that collective action, coalition partners and internal coordination are no longer needed. This also means that, in times of crisis, when renewed coordination is needed, but the network infrastructure has disappeared, more loose‐knit and unstable elite constellations are formed on the fly.^1^

**TABLE 1:** Elites by level and type of integration in the central circle of elite networks

		Cohesion (integration within central circle)	
		**Low**	**High**
**Sectoral bridging (Integration between groups in central circle)**	**Low**	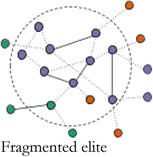	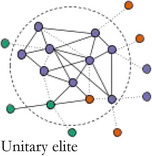
	**High**	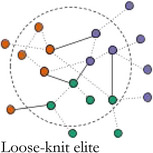	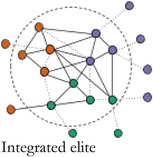

*Note*: Colors represent different sectors.

## 4 STRATEGY, DATA, AND METHODS

### 4.1 Building the Swiss elite networks and identifying the cores

This study relies on collaborative work resulting on a prosopographical database on Swiss elites constituted in the frame of research projects^2^ (the *Swiss elite database* ^3^ established by the Swiss Elite Observatory—OBELIS—at the University of Lausanne). The aim of this collection was to build a list of the most influential institutions, and the people sitting at their top, in key sectors of Swiss society, which could take into account historical changes during a period of more than a century. Once this list was established, with some organizations (e.g., companies) appearing or disappearing given their particular influence at a given time, a list of the people occupying top positions within these organizations was built (often comprising people sitting on the board of these organizations and thus occupying executive or supervisory positions, including the people running top public organizations and sitting in the federal parliament). Biographical information on selected individuals (gender, date of birth and death, citizenship, place of birth, marriage, and family background—when available—, education, main occupation, career information etc.) was collected as a collaborative process by the many researchers involved within the OBELIS team. In order to complement this collection, we proceeded to a more detailed prosopography on many biographical indicators once we had established the list of the members of the core (*k*‐shells) using a large variety of digital and historical sources and archives (some of these indicators being not featured in this present study, which addresses network and organizational dynamics, rather than biographical logics).

To study elite network changes, we rely upon an analytical strategy, which puts the focus on a thorough and theoretically‐informed description, rather than looking for the causes of elite changes. It follows a claim that descriptive work can be highly productive and should not be subordinated to explanatory claims. Description, and the related visual methods, allows researchers to focus on social and historical processes in order to enrich empirical qualitative and quantitative outcomes (Savage, 2009, 2020).^4^ Elite networks and their central social circles were built in a two‐step procedure. First, a broader set of elites was defined, according to their position at the top of the most influential institutions in eight sectors (business, unions, politics, administration, academia, expertise, other interest associations, and the military^5^) and were collected systematically for seven benchmark years. The network was built based on the idea that the elites that sit on the same organization at the same time know each other and interact within decision‐making bodies. Therefore, network ties not only mean connections between individuals and institutions (Gautier Morin & Rossier, 2021), but also among individuals who cultivate relations of knowledge, acquaintance, acknowledgement and interactions within important decision‐making processes that potentially impact Swiss society as a whole. As a result, we did not build the network of each individual during their entire career, but rather focused on their precise interactions with other elite members at a given benchmark year. These benchmarks were chosen to capture different historical logics: the end of the long 19th century and before World War I (1910), the interwar period (1937), the postwar period (1957), the period before (1980) and after (2000) financialization of the economy and autonomization of politics and academia, the direct aftermath of the financial crisis (2010), and the most recent period (2015). Second, through elites' name and institutional affiliations, we built two‐mode elite‐to‐affiliation networks for each year, using a total of 22,262 elite members tied to 2587 institutional affiliations. If two individuals were tied to the same affiliation at the same time, it means that they met personally and interacted by sitting together on its board or committee. We projected them into one‐mode elite‐to‐elite networks, for a total of 375,374 edges. We then applied a *k*‐shell decomposition procedure^6^ and identified for each cohort a central circle within the network for a total of 778 individual elite positions (738 individuals) tied by a total of 9856 edges. *K*‐shells indicate elite networks' cohesiveness. Sectoral diversity, based on the main sectoral occupation of shell members and the amount of sectors they were active in, is indicative of the level of sectoral bridging in elite networks. Table 2 summarizes the main properties of these networks. The mean k‐score (the average number of individuals, to which shell members are connected within their second neighborhood, *that is,* they know directly or indirectly through another shell member) is relatively high and stays more or less stable in proportion across time (if we divide it by the number of individuals in each shell), which we take as evidence of the historical robustness of the decomposition method.

**TABLE 2:** Properties of the networks

	1910	1937	1957	1980	2000	2010	2015
Total individuals	1584	1877	3110	4475	3738	3827	3651
*K*‐shell members	75	103	211	197	96	47	49
% *k*‐shells members	4.7%	5.5%	6.8%	4.4%	2.6%	1.2%	1.3%
Total edges	26,818	35,848	61,274	77,941	60,925	56,329	56,239
Edges among *k*‐shells	601	900	3991	2977	772	372	243
% *k*‐shells edges	2.2%	2.5%	6.5%	3.8%	1.3%	0.7%	0.4%
Density graph total	0.021	0.020	0.013	0.008	0.009	0.008	0.008
Density graph *k*‐shells	0.217	0.171	0.180	0.154	0.169	0.344	0.207
Mean *k*‐score	33	56	93	89	41	29	25

### 4.2 Indicators

Once the shells were identified, we collected indicators^7^ related to shell members' profiles. We look at the historical evolution of cohesiveness and sector affiliations of the core for each year through the share of *number of sectors*, *number of affiliations*, *affiliations to the main inter‐sectoral meeting places* (company boards, parliament, expert committees), *the main company subsector* and *political party affiliation*. We also look at the evolution of the most *recurring affiliations* and *main sector*, and comment on the cohesiveness and bridging in elite networks through *sectoral configurations* of the two‐mode *k‐shell networks*.

## 5 THE HISTORICAL EVOLUTION OF COHESIVENESS AND BRIDGING IN THE SWISS ELITE NETWORK’S CORE

We describe the evolution of network cohesiveness and bridging in terms of number of sectors and affiliations, recurring affiliations and main sector, then analyze the structure of the network’s core through our elite integration typology. Table 2 displayed changes in the core composition: from 1910 to 1980, the shells included between 4% and 7% of the elite population, but this share dropped significantly to 2% in 2000, and then 1% in 2010/2015. This drop is symptomatic of the fragmentation movement described earlier. Table 3 displays several features of the shells in terms of number of sectors and affiliations, ties to the main bridging organizations (company boards, the parliament, expert committees), company sector and political party affiliations.

**TABLE 3:** Sectoral composition of shells (in %)

Variable	1910	1937	1957	1980	2000	2010	2015
	%	%	%	%	%	%	%
Number of sectors							
1	29	21	7	12	22	21	27
2	41	49	34	41	45	60	53
3	19	20	41	36	32	19	16
4	11	9	16	10	1	0	2
5	0	1	2	2	0	0	2
Number of affiliations							
2	13	18	5	11	31	38	41
3	37	22	18	18	27	32	39
4	27	24	26	24	14	11	10
5	7	17	14	11	14	9	6
6	8	10	11	14	10	6	2
7	7	2	13	6	2	2	2
8	1	3	6	5	0	2	0
9+	0	3	9	10	2	0	0
Top 110 companies board							
Yes	99	95	58	38	49	6	84
Federal parliament							
Yes	33	15	24	14	8	9	2
Expert committee							
Yes	20	35	88	91	59	89	27
Main company subsector							
Banking and finance	60	52	20	14	30	2	31
Industry	39	43	34	17	16	4	45
Commerce	0	0	4	7	3	0	8
None	1	5	42	62	51	94	16
Political party							
Free Democrats/Liberals	44	35	23	23	26	17	10
Conservatives/Christian Democrats	8	4	12	12	7	9	4
People’s Party	0	2	7	7	1	9	4
Socialists/Greens	0	3	12	10	7	9	2
Other/elected without party affiliation	0	0	1	1	1	0	0
No known party affiliation	48	56	44	48	57	57	80
*N*	100% (75)	100% (103)	100% (211)	100% (197)	100% (96)	100% (47)	100% (49)

*Note*: Number of sectors: Sectors are the following (max. = 8): business; unions; politics; high civil service; expert committees; academia; associations; military officer. Main company subsector: In the case of affiliations to more than one company, the sector of the company with a CEO, chair or delegate of the board position prevails over the other positions as “simple” board member. Then, in the case of equal positions in more than one sector, a position in banking and finance prevails over a position in industry, and a position in industry prevails over a position in commerce.

**FIGURE 1:**
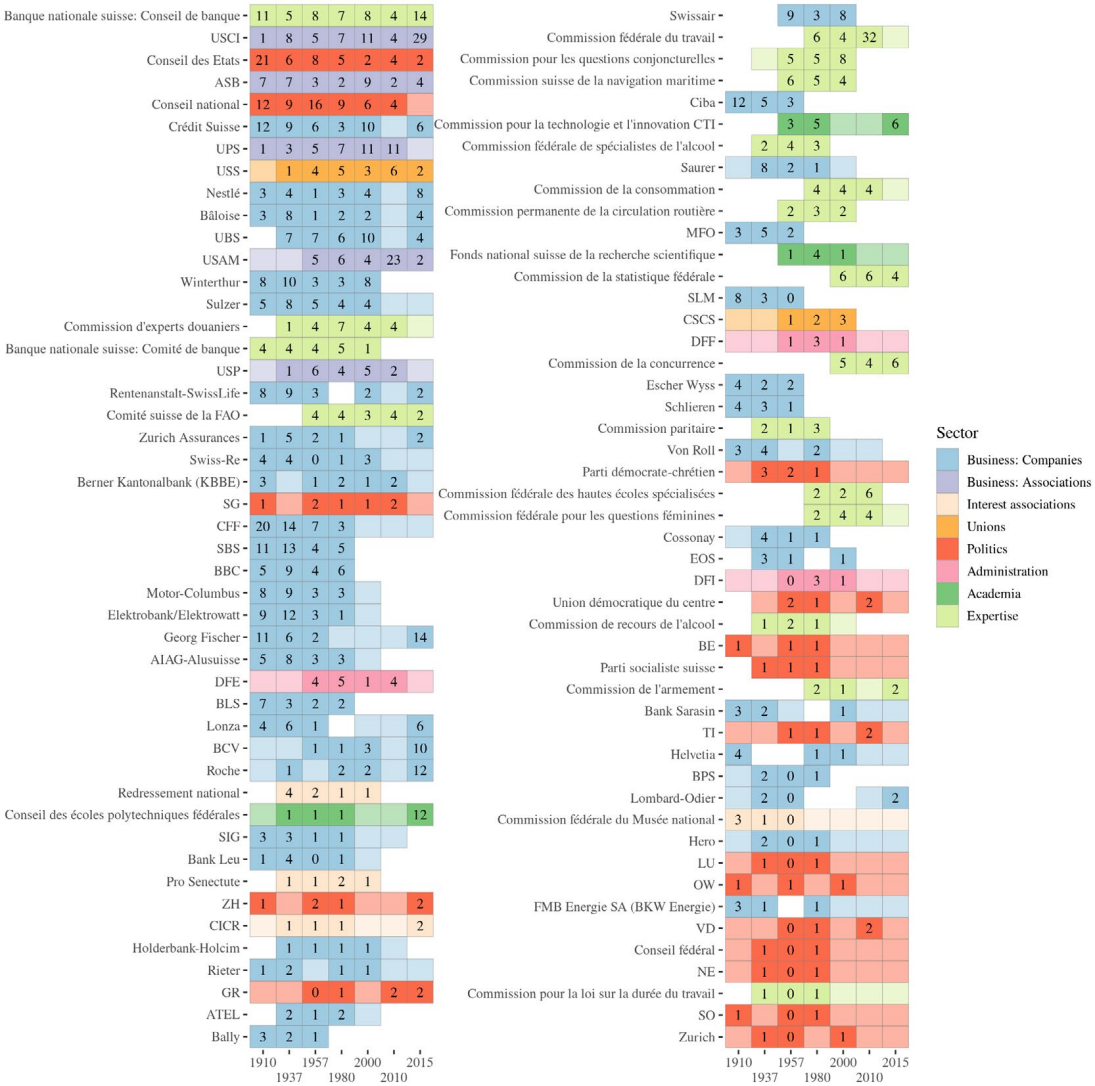
Heat map of recurring affiliations for at least 3 shell cohorts (in % of shell members affiliated) notes: Blank cells mean that the affiliation was not included for the year. Colored cells without a number mean that the affiliation was included, but no shell member was tied to it at the time

The core was the most *multisectorial* (59% of shell members were affiliated to at least 3 sectors in 1957, and 48% in 1980) and *multipositional* (affiliated to the largest number of organizations) in 1957 and 1980, during the elite “integration” period described earlier, while in 2010, following the 2008 financial crisis, the shells were affiliated to the smallest number of sectors. Among *bridging organizations* between sectors and positions, company boards (which can bridge business with other sectors, for example, by including politicians), both from the financial and industrial subsectors, were the most recurring during the elite “consolidation” period, but lost in importance after the 1930s and reached their lowest point (6%) after the financial crisis, when big companies had suffered a critical hit and were not able to play their bridging role. Nevertheless, in 2015, companies became again central. Following a somewhat opposite movement, expert committees increased during the integration period and lost their relevance only recently. The Parliament gradually became less central in bridging shell members, especially during the most recent “fragmentation” period, and political party affiliations followed a similar movement. The most important party was the Free Democratic Party, close to big companies and urban economic sectors, while links to the catholic Christian Democracy, the (at that time) agrarian Swiss People’s Party (formerly Party of Farmers, Traders and Independents) or the social‐democratic Swiss Socialist Party and their ally the Greens were equally marginal. In summary, in the consolidation period (1910, 1937), elites revolved primarily around company boards, while in the integration period (1957, 1980), elites relied more on neo‐corporatist committees. These organizations lost their prevalence again to company boards in the most recent year (2015). Figure 1 displays the organizations that bridged shell members for at least three cohorts.^8^

We observe some very stable bridging organizations: the two chambers of the Parliament, the five major business associations, one of the major unions (USS), the two non‐executive boards of the Central bank and some of the country’s most important companies: the largest banks (Crédit Suisse, UBS and SBS, that both merged into UBS in 1998) and insurances (Bâloise, Zurich Insurance, Winterthur, Swiss Re and SwissLife), the food company Nestlé, the manufacturing firm Sulzer or the pharmaceutical company Roche. Other companies were very important until the 1980s and then lost their centrality, *for example,* the state owned Swiss Federal Railways (SBB/CFF) or Swissair, which disappeared in the 2000s. State and political organizations were influential power brokers during the second part of the 20th century: expert committees (e.g.*,* regarding customs, economic forecasts or labour regulations), federal departments, canton and city governments and the main political parties. As already underlined, companies were the most bridging institutions in 1910 and 1937, then expert committees between 1957 and 2010, then companies again in 2015.^9^

**FIGURE 2:**
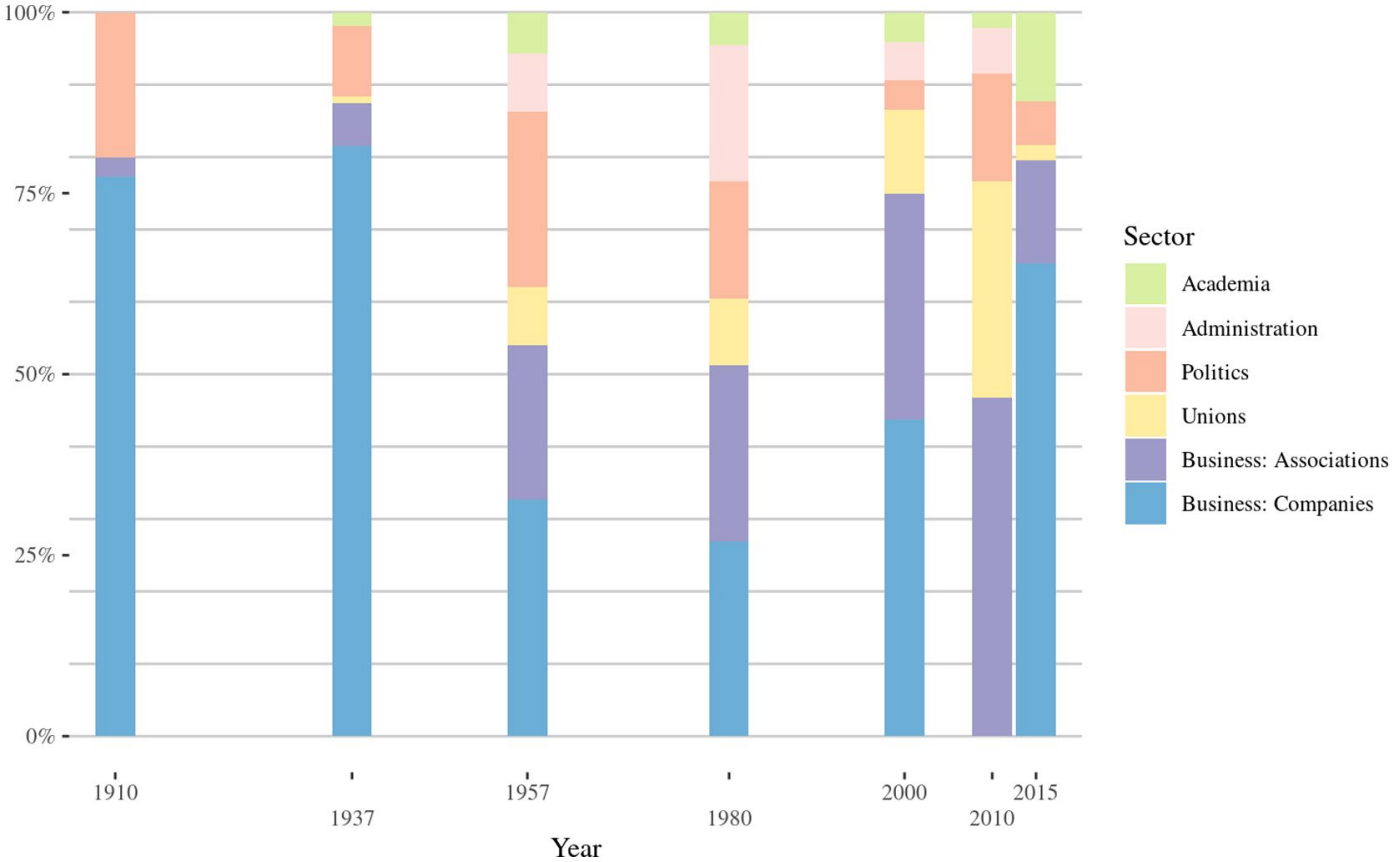
Evolution of the *k*‐shells 1910–2015, by main sector (in %)

**FIGURE 3:**
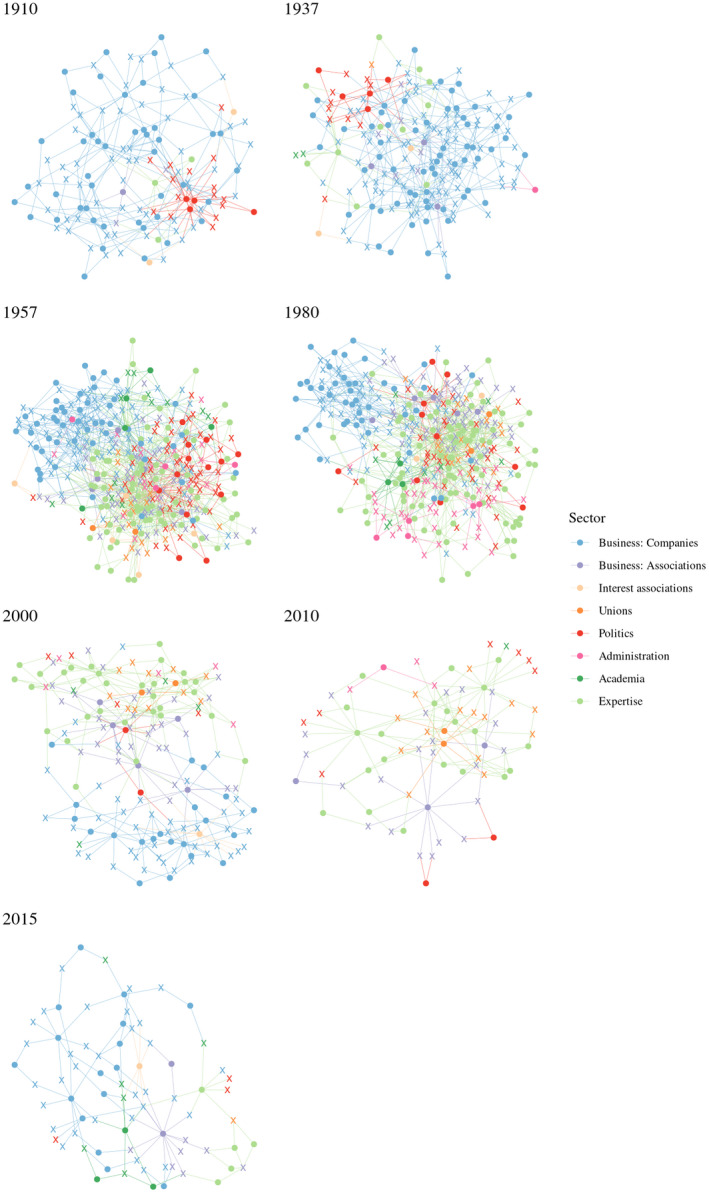
Two‐mode network graphs of *k*‐shells notes: Circles mark bridging affiliations and crosses mark core members. The colors correspond to the sector of the affiliation and the main sector of core members

Figure 2 displays the sectoral diversity according to the cores' main occupation (divided into five principal sectors: business, unions, politics, administration, academia).^10^ Business was always the dominant sector, especially during the elite consolidation period (1910, 1937) and again in 2000 and 2015 (elite fragmentation period).^11^ During the height of corporatism and elite integration (1957 and 1980), the core was more diverse and comprised more sectors: union leaders, politicians, civil servants and professors. State actors (political and administrative elites) were the most represented after business. Union elites acted for workers' interests in times of crisis; in the immediate aftermath of the financial crisis (2010), they became very present in the core, but were again excluded in 2015. Given this context, the core was more pluralistic again in 2010, since a relative weakening of business elites^12^ made union and political elites more integrated. In summary, elite cores were more integrative in 1957 and 1980, when the state coordinated the elites, and in 2010, right after the crisis, when, given the economic context, the state had to step in again. We now consider the cohesiveness and sectoral bridging levels of the seven cores by looking at elite network configurations. Figure 3 displays for each cohort the two‐mode networks of core members and the affiliations that bridged at least two of them.^13^

In 1910 and 1937, during the *consolidation period*, the core was highly cohesive, but the level of bridging between sectors was low. Companies and business associations constituted integrating affiliations of Swiss elites. Business elites were dominant, with each time a small group of somewhat peripheral political actors, who were integrated because of their proximity to business interests. This highly cohesive and lowly bridging *unitary elite core* enabled the corporate class to defend their interests, while expressing a limited commitment to other elite groups. After the Second War, during the *integration period*, a new elite constellation was in charge: in 1957 and 1980 the networks were highly cohesive and sectorally bridging. Core members stemmed from various sectors and sat on a variety of affiliations, among others bridging neo‐corporatist expert committees. This *integrated elite core* resembled the most to the power elite (Mills, 1956), a cross‐sectorial group with well‐established and long lasting alliances between key sectors.

After the 1980s, the elites followed a *fragmentation period*: the cores became less cohesive and connections between sectors diminished. In 2000, a less diversified core was separated into two sub‐groups: one group of business elites circling around companies, the other formed of a more heterogeneous crowd (politicians, unionists, civil servants, professors) organized around expert committees, while political organizations and business associations bridged between the two. In 2010, the core became very small, but more pluralistic than in 2000. It was formed of politicians and union and business association leaders tied to central expertise committees. In 2015, sectoral diversity was the lowest since 1937, with overwhelmingly present business elites who mostly organized around companies. Because of a renewed hegemony of business and a lesser need for collective action after the 1980s, the central group tended to correspond to a *fragmented elite core* in 2000 and 2015, with a low internal cohesion and low integration between sectors. However, in 2010, it corresponded to an unstable *loose‐knit elite core*, still with a low integration, but a higher bridging between sectors, as a response to the short‐term need for a renewed coordination in the crisis context, but without a strong internal integration. In summary, following historical developments (i.e.*,* the aftermath of World War 2, the financialization process of the late 20th century and the 2008 crisis) and along with different affiliation configurations, the Swiss elite core changed drastically, from unitary elites to more integrated ones, and finally to more fragmented or loose‐knit elite versions. The renewed importance of business since the 1990s echoes Savage’s (2021) argument of a return to a past order, where wealth inequality is as important in the recent period as it was before the First World War. At the same time, the recent fragmentation of the elites goes in line with his description of a process of “entropy” in social fields, which become less autonomous, less cohesive and more permeable, while wealth and other forms of economic capital, accumulated by a small group of individuals organized around the most powerful companies, reassert themselves once again as the most distinctive form of capital in society.

## 6 DISCUSSIONS AND CONCLUSIONS

We observed historical evolutions of the Swiss elites' core during 105 years, based on a typology of network cohesion and sectoral bridging in Switzerland over time. Table 4 summarizes our main findings.

**TABLE 4:** Typology of the Swiss elite core between 1910 and 2015

		Cohesion (integration within central circle)	
		Low	High
**Sectoral bridging (Integration between groups in central circle)**	**Low**	Fragmented elite core	Unitary elite core
		Years: 2000, 2015 (“fragmentation” period)	Years: 1910, 1937 (“consolidation” period)
		Characteristics: Loosely integrated business elites around companies and other organizations	Characteristics: Well‐integrated business elites
	**High**	Loose‐knit elite core	Integrated elite core
		Year: 2010 (“fragmentation” period)	Years: 1957, 1980 (“integration” period)
		Characteristics: Loosely integrated multisectorial elites around expert committees	Characteristics: Well‐integrated elites from a diversity of sectors around expert committees

We found that the core was always dominated by business, business organizations and corporate forms of legitimacy, but at historical times of crisis to the hegemony of business elites, *that is,* after World War 2 with the increase in neo‐corporatist processes, and shortly after the 2008 financial crisis with the turmoil that multinational companies and banks went through, elite circles expanded and the core included individuals with delegated forms of power (politicians, civil servants, unionists, university professors). We found that, during the *elite consolidation period* (1910, 1937), the core was composed of a highly cohesive corporate *unitary elite*. Then, during the *elite integration period* (1957, 1980), the core moved toward a corporatist *integrated elite*, stemming from a diversity of sectors and organized around several types of affiliations, but most of all state expert committees. Finally, during the *fragmentation period* (2000, 2010, 2015), the cores became less cohesive, coherent, and organized. We found two elite configurations during the most recent period. In 2000 and 2015, the core took the form of a *fragmented elite*, composed of a loosely integrated group of (mostly) business elites organized around companies and other types of affiliations. In 2010, after the financial crisis, the core took the shape of a *loose‐knit elite*, formed of loosely integrated pluralist elites from a variety of sectors organized again around neo‐corporatist committees.

These results were obtained by looking at the changes of a network that reunites the most important elite groups from each key sector in Switzerland, while being sensitive to historical and institutional dynamics. We identified the cores through a robust method already used on other elite networks (Larsen & Ellersgaard 2017). Our findings show the potential of analyzing the evolution in elite networks during a long period of time. Some changes could be caused by the varying reliability of our historical information in ties to key affiliations by elites, making the core group appear less stable and have less continuity. When some key institutions were not included in the core for a certain period of time (for example most companies in 2010), it does not mean that they disappeared from the network, but rather that they occupied a more peripheral position, which has to be interpreted in terms of distance to the core.^14^ While we lack year‐to‐year granularity of the data, the findings still point to a particular development of Swiss elites. These dynamics merit exploration in other cases as well, comparing historical periods, but also engaging in a cross‐country comparison, to focus on various elite network strategies when facing different types of pressure from below.

As a small capitalist state, the role of business in elite networks in Switzerland is of particular interest. In the start of the period, corresponding to the gilded age of high concentration of income and wealth described by Piketty (2020), we observed an inner‐circle‐strategy of business at the core of the network. Cohesion in the corporate network was high with key alliances to most important state officials, making the corporate elites able to act as a class‐for‐itself *vis‐à‐vis* other elite groups. Post‐World War 2, the core of the elite network diversified to include key actors from politics, the state and unions, coinciding with periods of welfare state development and lower levels of economic inequality. However, as also shown by Mizruchi (2013) in the US case, this network fragmented as neoliberalism developed and inequality levels were again on the rise. Unlike in the early part of the 20th century, this movement did not mean a return to strong intra‐corporate networks. Rather, the political success of neoliberalism reduced the demand for intra‐corporate cohesion leading to a situation in which one gets what one wants, but has no social infrastructure to decide what one wants. This made the rapid changes in the 2010 network core of the Swiss elite networks noteworthy. In a situation of acute legitimacy crises of Swiss business, key actors from other sectors were suddenly called upon to restore a broader social alliance behind Swiss capitalism. Finally, in the most recent period, although business elites are less integrated, the prevalence of corporate elites and their accumulated wealth and economic capital has returned to the level of 1910, as described by Savage (2021), following the findings of Piketty (2014, 2020)

These changes in elite cohesion were accompanied in Switzerland by diverse economic and structural changes related to new exercise of elite power. For example, during the 1990s, at the beginning of the elite fragmentation period, new elite groups were able to converge. Liberal economics professors, allegedly marginalized from expert committees, formed an alliance with an informal group of big company representatives, outside of the traditional business association channels. Together, they issued various documents constituting a program “for a more liberal order” with the aim to liberalize domestic markets, privatize the major public utilities and reduce fiscal pressures. Their action was supported by the influential think tank *AvenirSuisse*. This “neo‐liberal coalition” succeeded in being heard by the Swiss government, who appointed a special commission composed of their main members and without any union representative, paving the way for some liberal reformative agenda in the 1990s (Mach, 2002). This coalition took advantage of the fracturing logics of elites to become influential through new (more formal and public) channels, therefore relying less on network connections to do so (Mach et al., 2021). More generally, these dynamics were accompanied by several changes in Swiss society, such as an increasing transnationalization of the Swiss economy and labour markets,^15^ an increase in income and wealth inequality at the very top (Martinez, 2017) or a decentralization and deregulation of collective bargaining (Mach & Oesch, 2003).

This analysis of the level of cohesion and cross‐sectoral bridging in elite networks in Switzerland across 105 years has shown the different alliances forged by big business to defend their interest: From a starting point of business unity, but little ability to bridge toward other sectors, followed by a new deal‐like post‐World War II coalition with labour, politics, state, and academia to a neoliberal era in which business relies more on their structural power than network embeddedness to protect their interest. However, once the legitimacy is threatened, as in 2010 after the financial crisis, which hit cornerstones at the top of the Swiss corporate elite particularly hard, the cross‐sectoral bridges are reactivated, albeit only temporarily. This possible relationship between legitimacy strategies of capital, developments in inequality and the structure and composition of elite networks call for more discussion of the relation between the sociology of elite networks and comparative political economy (for a cross‐sectional perspective, see: Cárdenas, 2012). Two lines of inquiry, both inspired by neo‐gramscian perspectives, seem particularly promising. First, exploring to what extent elite networks mirror social blocs behind particular growth models in the perspective outlined by Baccaro and Pontusson (2019) could help us understand the economic underpinnings of elite networks. Second, exploring if and how different elite network constellations draw on development of hegemonic projects (Jessop, 2016) could aid our explorations of how the ideological foundation of a changing elite is maintained. The development in the core of the Swiss elite networks analyzed here certainly suggests that the structure of elite networks reflects the balance between social forces and gives us a blueprint to understand the strength of key forms of power in society. To conclude our paper, this research also opened on promising directions for the study of elite cohesion. Elite integration does not only happen through organizational ties, but also through common profile features, related to different forms of assets and resources, whether ascriptive, and linked to social background, elite family ties, gender and race or ethnicity, or acquired, and related to educational and professional features. In particular, it would be relevant to study changes in the core in the light of Bourdieu’s (1996) description of the historical evolution of elite reproduction between family‐based and educational‐based logics. The account of these changes could be a first step into the historical study of the evolution of the strength of different forms of (economic, cultural and social) capital throughout the 20th century by considering the members of this core as “effective agents” within the field of power (Lunding et al., 2021).

## Supporting information


Appendix

## Data Availability

Data sharing is not applicable to this article as no new data were created or analyzed in this study.

## ENDNOTES

^1^
In this model, we did not take into account how these elite groups acquired, accumulated and converted different forms of capital and resources—including network assets, that is, social capital—and how different elite fractions related to each other based upon their network embeddedness within the Swiss field of power (Bourdieu, 1996). This is part of an ongoing investigation that will lead us to other writings on the topic. The concept of the field of power is particularly relevant to study the evolution of the structure of the core of the network, including the recent fragmentation of elite relations and the resulting contestation of elite order due to these mechanisms.

^2^
The main projects, funded by the Swiss National Science Foundation, were: “Les réglementations du gouvernement des entreprises en Suisse: origine, évolution et changements récents du comportement des entreprises” (PI: Thomas David; 2003–2006); “Les élites suisses au 20e siècle: un processus de différenciation inachevé?” (PIs: André Mach and Thomas David; 2007–2010); “Academic Elites in Switzerland 1910–2000: Between Autonomy and Power” (PIs: Felix Bühlmann, André Mach and Thomas David; 2013–2017); “The Swiss Power Elite and Field of Power. Tensions between Elite Coordination and Differentiation since the 1950s” (PI: Thierry Rossier, 2019–2020). Archival and historical work was undertaken to obtain the list of relevant organizations and individuals. The main sources to establish this list were the following: the commerce register and the economic and financial press for the business and union sectors; the website of the Swiss parliament and other cantonal websites and press resources for the political sector; the digitalized archives of the federal administration for the administrative and the military sectors; university personnel directories for the academic sector; and other annual reports and publicly available registers for other associations and organizations. See the details of the projects and used sources: https://www.unil.ch/obelis/en/home/menuinst/projects.html.

^3^
See the website of the Swiss Elite Database, here: https://www2.unil.ch/elitessuisses.

^4^
A descriptive approach can be obviously complemented by other types of analysis, such as statistical modeling. In particular, exponential random graph models (ERMGs) could help us to model network dynamics more deeply, according to micro‐level individual and institutional mechanisms. However, this type of analysis is beyond the scope of this precise paper and should be explored in another study.

^5^
See the details of the retained organizations for each sector in Table A of Online Appendix 1.

^6^
See the description of the procedure in Online Appendix 2.

^7^
See the details of the different used indicators in Online Appendix 3.

^8^
In this table, most of the names of the affiliations are given in French. This should not be interpreted as a dominance of French‐speaking institutions at the expense of other linguistic regions. Indeed, as the Swiss elite team is mostly based in French‐speaking universities, the data were collected in French, and that included the names of elite organizations at the federal level, as featured in this study. However, these organizations are normally named differently in every official language. For example, the Swiss Federal Railways company is called CFF (*Chemins de fer fédéraux suisses*) in French, SBB (*Schweizerische Bundesbahnen*) in German, FFS (*Ferrovie federali svizzere*) in Italian and *Viafiers federalas svizras* in Romansh. More generally, an ongoing investigation on the geographical profile of the core members that will be featured in another study reveals that their distribution in terms of linguistic regions mostly matches the current distribution of the Swiss population.

^9^
See in Tables B to H in Online Appendix 4 the 20 most recurring affiliations in the k‐shells for each year. See also Tables I and J in Online Appendix 5 for the details of the economic subsector for all the retained companies and for all the companies linked to at least one member of the k‐shells. We see that, to the exception of 2010, where only three companies were linked to the core, the representation of the different economic subsectors stayed quite stable for the whole period.

^10^
See Online Appendix 6 for the details of the positions of shell members by sector (Table K) and of their multisectoriality (Table L).

^11^
We actually split business into two subsectors, companies and associations, to let visible the evolution between the two.

^12^
Corporate actors were completely excluded from the 2010 core, but were replaced by business association leaders, who acted in favor of business interests through neo‐corporatist expert committees.

^13^
See Online Appendix 7 for the two‐mode graphs of the whole largest component.

^14^
See again the complete networks in Online Appendix 7 to make sense of what the distance to the core means for each sector at each benchmark year.

^15^
These transnationalization logics obviously had an impact on the profile of the members of the core, which followed the same evolution of the U‐curve displayed in Figure 2: during the consolidation period (1910 and 1937) and for the most recent cohort (2015), there were more non‐Swiss members of the core and more individuals had studied or worked outside Switzerland for a significant period of time than during the integration period. These results will be investigated further in another study.
