# Supplementary material for: From integrated to fragmented elites. The core of Swiss elite networks 1910–2015
Source: Br J Sociol. 2022 Feb 14;73(2):315–35. doi: 10.1111/1468-4446.12929 (PMC9305851; doi:10.1111/1468-4446.12929)
Supplement: Supplementary file 1 — Appendix [file BJOS-73-315-s001.docx]

***Online appendix 1: Types of affiliations in the network***

*Table A: Types of affiliations in the network (absolute numbers)*

| **Elites** | **Type of organisations** | **1910** | **1937** | **1957** | **1980** | **2000** | **2010** | **2015** |
| --- | --- | --- | --- | --- | --- | --- | --- | --- |
| Academia | Associations and academic organisations | 4 | 4 | 10 | 10 | 11 | 11 | 11 |
|  | Universities | 8 | 9 | 9 | 10 | 12 | 12 | 12 |
| Administration | Supreme court | 1 | 1 | 1 | 1 | 1 | 1 | 1 |
|  | Central bank | 1 | 1 | 1 | 1 | 1 | 1 | 1 |
|  | Federal departments | 8 | 8 | 8 | 8 | 8 | 8 | 8 |
| Expertise | Expert committees | 58 | 72 | 214 | 298 | 183 | 216 | 143 |
| Business | Top companies | 112 | 110 | 108 | 107 | 112 | 108 | 109 |
|  | Main business associations | 5 | 5 | 5 | 5 | 5 | 5 | 5 |
| Unions | Main unions | 2 | 2 | 2 | 2 | 2 | 2 | 2 |
| Politics | Federal government | 1 | 1 | 1 | 1 | 1 | 1 | 1 |
|  | Federal parliament | 2 | 2 | 2 | 2 | 2 | 2 | 2 |
|  | Cantonal governments | 25 | 25 | 25 | 26 | 26 | 26 | 26 |
|  | Largest city governments | 4 | 4 | 4 | 4 | 4 | 4 | 4 |
|  | Main political parties | 2 | 4 | 4 | 4 | 5 | 6 | 7 |
| Interest associations | Most influential associations and organisations | 14 | 20 | 20 | 23 | 23 | 9 | 9 |
| Military | The generals | 1 | 1 | 1 | 1 | 1 | 1 | 1 |
| **Total** |  | **248** | **269** | **415** | **503** | **397** | **413** | **342** |

Elite individuals were divided into eight main categories according to the sector of the institutions they were connected to (one individual can be affiliated to more than one sector or type of organisation). *Academic elites* unite the executive board members of the main scientific associations and academic organisations, as well as all the rectors/presidents and department deans of each cantonal university and federal technical school. *Administrative elites* relate to federal judges (Supreme court members), Swiss National Bank (*i.e.* the Swiss central bank) directors as well as, in the seven federal departments and the federal chancellery, the seven elected ministers (federal councillors), the chancellor and vice chancellors, the general secretaries of each department and the directors of each federal office. *Expertise elites* correspond to all expert committee (extra-parliamentary commissions) members for the Federal administration. *Business elites* include the CEOs, delegates of the board and other (non-executive) board members of the largest *ca.* 110 Swiss companies (according to their turnover, number of employees, assets and market capitalisation^[[1]](#footnote-1)^), and the executive committee members of the five most important business associations. *Union elites* comprise the executive committee members of the two most influential trade unions. *Political elites* are composed of the seven members of the federal government (federal councillors), the members of the two chambers of the federal parliament, the members of all the cantonal governments, the members of the governments of the largest cities (Zurich, Geneva, Bern and Lausanne; the governments of the city and the canton of Basel are actually the same institution) and the executive committee members of the main political parties. *Interest associations* (other than business associations and unions) are formed by the board members of other main associations, foundations, societies, NGOs or think tanks, which do not enter in any other categories. Finally, in the *military elites* are retained the highest ranking generals (*Korpskommandanten*).

***Online appendix 2: K-shell decomposition***

The k-shell procedure used in this paper identifies the densest, most cohesive and central part of the network and is a modification of the method proposed in Larsen & Ellersgaard (2017)^[[2]](#footnote-2)^. The one-mode projection of the affiliation network is weighted according to the number of members in each affiliation above 14, so that larger affiliations create weaker ties and the strongest multiple ties are weighted down. The precision of k-shell and the k-core decomposition can be improved by first pruning redundant ties^[[3]](#footnote-3)^. The network is then pruned so that it only includes nodes that create unique ties in the largest component. This is done by iteratively removing nodes with a local betweenness score below their weighted degree. The pruning procedure ensures that nested or very strongly overlapping affiliations are not unduly influencing the core definition. After the initial pruning, we apply a k-shell algorithm to a reach graph, where all nodes are connected to their neighborhood. The neighborhood threshold is varied between path lengths of 2 and 2.25 in order to reduce the importance of the threshold. Individuals in the highest k-shell in more than 50% of the 26 shells are the core for that given period. The k-shell^[[4]](#footnote-4)^ is conceptually similar to the k-core^[[5]](#footnote-5)^ but works on a weighted matrix instead of a binary and, since the network is weighted, this is a better fit. But results between the two variations overlap substantially.

***Online appendix 3: Details of the used variables***

To describe the historical evolution of sector affiliations of the Swiss elite network’s core, we use the following indicators:

- **The number of sectors** is measured by the total number of sectors covered at the time through elite affiliations over a total of seven sectors: business, unions, politics, administration, academia, expert committees, and other types of interest associations. We then add an eighth sector based on whether or not they were officers in the Swiss military at the time (rank between lieutenant and general).
- **The number of affiliations** is measured by the total number of elite affiliations at the time.
- **Affiliations to the main inter-sectoral meeting places** are assessed through whether or not they sat on the *board of the 110 largest companies*, the *federal parliament* and *expert committees* at the time.
- **The main company sector** is assigned to the following categories: banking and finance, industry, commerce, or none (in the case of no affiliation to the board of one of the largest 110 companies). In the case of multiple company affiliations, the sector of the company with a CEO, chair or delegate position prevails over the other board member positions. In the case of affiliations to more than one sector, banking and finance prevails over industry, and industry prevails over commerce.
- **Political party affiliation** is assessed through a known affiliation to one of the main political parties.
- **The most recurring affiliations** are assessed by the number of shell members sitting on an affiliation, which appears for at least three of the seven elite cohorts.
- **The main sector** over time is based on the main sectoral occupation of shell members. We divide them into five categories: business (companies and associations), unions, politics, administration and academia (none of them being affiliated to expertise, civil society or the military as primary sector).

***Online appendix 4: Most recurring affiliations***

*Table B: 20 most central organisations in k-shells in 1910*

| **Rank** | **Affiliation** | **N** | **Sector** |
| --- | --- | --- | --- |
| 1 | Conseil des Etats | 16 | Parliament |
| 2 | CFF | 15 | Company |
| 3 | Anlagewerte | 9 | Company |
| 3 | Ciba | 9 | Company |
| 3 | Conseil national | 9 | Parliament |
| 3 | Crédit Suisse | 9 | Company |
| 7 | Banque nationale suisse: Conseil de banque | 8 | Expert committee (Central Bank council) |
| 7 | Georg Fischer | 8 | Company |
| 7 | SBS | 8 | Company |
| 10 | Alioth | 7 | Company |
| 10 | Elektrobank/Elektrowatt | 7 | Company |
| 10 | FM Beznau | 7 | Company |
| 13 | Bank in Winterthur | 6 | Company |
| 13 | Metallwerte | 6 | Company |
| 13 | Motor-Columbus | 6 | Company |
| 13 | Rentenanstalt-SwissLife | 6 | Company |
| 13 | SLM | 6 | Company |
| 13 | Winterthur | 6 | Company |
| 19 | ASB | 5 | Business association |
| 19 | BLS | 5 | Company |
| 19 | Buss | 5 | Company |
| 19 | EL Olten-Aarburg | 5 | Company |
| 19 | Parti radical-démocratique suisse | 5 | Political party |

*Table C: 20 most central organisations in k-shells in 1937*

| **Rank** | **Affiliation** | **N** | **Sector** |
| --- | --- | --- | --- |
| 1 | CFF | 14 | Company |
| 2 | SBS | 13 | Company |
| 3 | Commission fédérale de l'économie hydraulique | 12 | Expert committee |
| 3 | Elektrobank/Elektrowatt | 12 | Company |
| 5 | SFIS | 11 | Company |
| 6 | Winterthur | 10 | Company |
| 7 | BBC | 9 | Company |
| 7 | Conseil national | 9 | Parliament |
| 7 | Crédit Suisse | 9 | Company |
| 7 | Elektrowerte | 9 | Company |
| 7 | Motor-Columbus | 9 | Company |
| 7 | Rentenanstalt-SwissLife | 9 | Company |
| 13 | AIAG-Alusuisse | 8 | Company |
| 13 | Bâloise | 8 | Company |
| 13 | Saurer | 8 | Company |
| 13 | Sulzer | 8 | Company |
| 13 | USCI | 8 | Business association |
| 18 | ASB | 7 | Business association |
| 18 | UBS | 7 | Company |
| 20 | Conseil des Etats | 6 | Parliament |
| 20 | Georg Fischer | 6 | Company |
| 20 | Lonza | 6 | Company |

*Table D: 20 most central organisations in k-shells in 1957*

| **Rank** | **Affiliation** | **N** | **Sector** |
| --- | --- | --- | --- |
| 1 | Conseil national | 34 | Parliament |
| 2 | Comité consultatif pour les questions conjoncturelles | 27 | Expert committee |
| 3 | Commission fédérale de l'énergie atomique | 21 | Expert committee |
| 4 | Commission fédérale de l'assurance-vieillesse et survivants | 20 | Expert committee |
| 5 | Commission consultative de la politique commerciale | 18 | Expert committee |
| 5 | Swissair | 18 | Company |
| 7 | Banque nationale suisse: Conseil de banque | 17 | Expert committee (Central Bank council) |
| 7 | Commission des cartels | 17 | Expert committee |
| 9 | Comité professionnel de la viande | 16 | Expert committee |
| 9 | Conseil des Etats | 16 | Parliament |
| 11 | CFF | 15 | Company |
| 11 | Commission fédérale d'experts pour l'introduction de l'assurance invalidité | 15 | Expert committee |
| 11 | Commission fédérale de l'économie hydraulique | 15 | Expert committee |
| 11 | Commission pour la lutte contre les crises et la création d'emploi | 15 | Expert committee |
| 11 | UBS | 15 | Company |
| 16 | Commission fédérale pour les questions intéressant le marché de l'emploi | 14 | Expert committee |
| 17 | Commission consultative pour l'exécution de la loi sur l'agriculture | 13 | Expert committee |
| 17 | Commission du contrôle des prix | 13 | Expert committee |
| 17 | Crédit Suisse | 13 | Company |
| 20 | Fonds de compensation de l'AVS Conseil d'administration | 13 | Expert committee |

*Table E: 20 most central organisations in k-shells in 1980*

| **Rank** | **Affiliation** | **N** | **Sector** |
| --- | --- | --- | --- |
| 1 | Commission consultative de la politique commerciale | 26 | Expert committee |
| 2 | Commission fédérale des possibilités de travai | 22 | Expert committee |
| 2 | Commission pour la lutte contre les crises et la création d'emploi | 22 | Expert committee |
| 4 | Commission fédérale de l'assurance-vieillesse et survivants | 21 | Expert committee |
| 5 | Conseil national | 18 | Parliament |
| 6 | Commission consultative pour le développement économique régional | 16 | Expert committee |
| 6 | Commission fédérale pour les questions intéressant le marché de l'emploi | 16 | Expert committee |
| 8 | Banque nationale suisse: Conseil de banque | 14 | Expert committee (Central Bank council) |
| 8 | Fonds de compensation de l'AVS Conseil d'administration | 14 | Expert committee |
| 8 | USCI | 14 | Business association |
| 11 | Commission consultative pour l'exécution de la loi sur l'agriculture | 13 | Expert committee |
| 11 | Commission d'experts douaniers | 13 | Expert committee |
| 13 | UPS | 13 | Business association |
| 14 | Commission de surveillance du fonds de compensation de l'assurance-chômage | 12 | Expert committee |
| 14 | Commission fédérale du travail | 12 | Expert committee |
| 14 | Groupe de travail: "Politique structurelle" | 12 | Expert committee |
| 17 | BBC | 11 | Company |
| 17 | Commission du contrôle des prix | 11 | Expert committee |
| 17 | Forum chargé d'examiner les questions que pose le secteur des constructions | 11 | Expert committee |
| 17 | UBS | 11 | Company |
| 17 | USAM | 11 | Business association |

*Table F: 20 most central organisations in k-shells in 2000*

| **Rank** | **Affiliation** | **N** | **Sector** |
| --- | --- | --- | --- |
| 1 | Commission de surveillance du fonds de compensation de l'assurance-chômage | 15 | Expert committee |
| 2 | Avenir suisse | 12 | Think tank |
| 2 | Commission consultative pour l'exécution de la loi sur l'agriculture | 12 | Expert committee |
| 4 | UPS | 11 | Business association |
| 4 | USCI | 11 | Business association |
| 6 | Crédit Suisse | 10 | Company |
| 6 | UBS | 10 | Company |
| 8 | ASB | 9 | Business association |
| 8 | Commission consultative de la politique commerciale | 9 | Expert committee |
| 8 | Commission fédérale de l'assurance-vieillesse et survivants | 9 | Expert committee |
| 8 | Conseil d'administration de la Caisse nationale suisse d'assurance en cas d'accident | 9 | Expert committee |
| 12 | Banque nationale suisse: Conseil de banque | 8 | Expert committee (Central Bank council) |
| 12 | Commission fédérale de la prévoyance professionnelle | 8 | Expert committee |
| 12 | Commission pour les questions conjoncturelles | 8 | Expert committee |
| 12 | Swissair | 8 | Company |
| 12 | Winterthur | 8 | Company |
| 17 | Fondation Fonds de garantie LPP Conseil de fondation | 7 | Expert committee |
| 18 | Commission de la statistique fédérale | 6 | Expert committee |
| 18 | Conseil national | 6 | Parliament |
| 18 | Fonds de compensation de l'AVS Conseil d'administration | 6 | Expert committee |

*Table G: 20 most central organisations in k-shells in 2010*

| **Rank** | **Affiliation** | **N** | **Sector** |
| --- | --- | --- | --- |
| 1 | Commission fédérale du travail | 15 | Expert committee |
| 2 | Commission de la politique économique | 12 | Expert committee |
| 3 | USAM | 11 | Business association |
| 4 | Commission tripartite fédérale pour les mesures d'accompagnement à la libre circulation des personnes | 10 | Expert committee |
| 5 | Conseil d'administration de la Caisse nationale suisse d'assurance en cas d'accident | 9 | Expert committee |
| 6 | Commission de surveillance du fonds de compensation de l'assurance-chômage | 8 | Expert committee |
| 7 | Travail.Suisse | 7 | Union |
| 8 | Commission fédérale tripartite pour les affaires de l'OIT | 6 | Expert committee |
| 9 | UPS | 5 | Business association |
| 10 | Caisse supplétive LAA Conseil de fondation | 4 | Expert committee |
| 10 | Commission fédérale de l'assurance-vieillesse et survivants | 4 | Expert committee |
| 10 | Commission fédérale de la prévoyance professionnelle | 4 | Expert committee |
| 10 | Fondation Fonds de garantie LPP Conseil de fondation | 4 | Expert committee |
| 10 | Fonds de compensation de l'AVS Conseil d'administration | 4 | Expert committee |
| 15 | Commission de la statistique fédérale | 3 | Expert committee |
| 15 | Commission fédérale des hautes écoles spécialisées | 3 | Expert committee |
| 15 | USS | 3 | Union |
| 18 | Banque nationale suisse: Conseil de banque | 2 | Expert committee (Central Bank council) |
| 18 | Comité suisse de la FAO | 2 | Expert committee |
| 18 | Commission d'experts douaniers | 2 | Expert committee |
| 18 | Commission de la concurrence | 2 | Expert committee |
| 18 | Commission de la consommation | 2 | Expert committee |
| 18 | Commission fédérale de la formation professionnelle | 2 | Expert committee |
| 18 | Commission fédérale pour les questions de migration (CFM) | 2 | Expert committee |
| 18 | Commission fédérale pour les questions féminines | 2 | Expert committee |
| 18 | Conseil des Etats | 2 | Parliament |
| 18 | Conseil national | 2 | Parliament |
| 18 | DFE | 2 | Federal department |
| 18 | USCI | 2 | Business association |

*Table H: 20 most central organisations in k-shells in 2015*

| **Rank** | **Affiliation** | **N** | **Sector** |
| --- | --- | --- | --- |
| 1 | USCI | 14 | Business association |
| 2 | Avenir suisse | 7 | Think tank |
| 2 | Banque nationale suisse: Conseil de banque | 7 | Expert committee (Central Bank council) |
| 2 | Georg Fischer | 7 | Company |
| 2 | Givaudan | 7 | Company |
| 6 | Conseil des écoles polytechniques fédérales | 6 | Expert committee (Academic organisation) |
| 6 | Roche | 6 | Company |
| 8 | BCV | 5 | Company |
| 9 | Nestlé | 4 | Company |
| 10 | Commission de la concurrence | 3 | Expert committee |
| 10 | Commission pour la technologie et l'innovation CTI | 3 | Expert committee (Academic organisation) |
| 10 | Coop Group Cooperative | 3 | Company |
| 10 | Crédit Suisse | 3 | Company |
| 10 | Lonza | 3 | Company |
| 10 | Mobiliar | 3 | Company |
| 10 | Syngenta | 3 | Company |
| 17 | Ammann | 2 | Company |
| 17 | ASB | 2 | Business association |
| 17 | Bâloise | 2 | Company |
| 17 | Commission de la statistique fédérale | 2 | Expert committee |
| 17 | Commission fédérale consultative du Point de contact national pour les Principes directeurs de l'OCDE à l'intention des entreprises multinationales | 2 | Expert committee |
| 17 | EPFZ | 2 | University |
| 17 | Fonds de compensation de l'AVS Conseil d'administration | 2 | Expert committee |
| 17 | Implenia | 2 | Company |
| 17 | Kaba | 2 | Company |
| 17 | Schindler | 2 | Company |
| 17 | UBS | 2 | Company |

***Online appendix 5: Details of the economic subsectors of the retained companies***

*Table I: Detailed economic subsector for all the retained companies*

| **Subsector** | **1910** | **1937** | **1957** | **1980** | **2000** | **2010** | **2015** |
| --- | --- | --- | --- | --- | --- | --- | --- |
| Food and tobacco | 11% (13) | 6% (7) | 8% (9) | 10% (11) | 2% (3) | 6% (7) | 6% (7) |
| Insurance | 7% (8) | 7% (8) | 7% (8) | 9% (10) | 6% (7) | 7% (8) | 8% (9) |
| Banks and financial companies | 23% (26) | 22% (25) | 17% (19) | 17% (19) | 17% (20) | 14% (16) | 15% (17) |
| Chemicals | 4% (5) | 6% (7) | 6% (7) | 4% (5) | 9% (11) | 9% (10) | 9% (10) |
| Construction: cement | 0% (0) | 1% (2) | 1% (2) | 3% (4) | 5% (6) | 4% (5) | 4% (5) |
| Distribution of durable and non-durable goods, food shops | 0% (1) | 1% (2) | 6% (7) | 9% (10) | 8% (10) | 5% (6) | 4% (5) |
| Energy | 4% (5) | 6% (7) | 4% (5) | 4% (5) | 5% (6) | 4% (5) | 4% (5) |
| Watchmaking | 2% (3) | 5% (6) | 2% (3) | 0% (1) | 1% (2) | 2% (3) | 2% (3) |
| Equipment: wood, paper | 1% (2) | 2% (3) | 2% (3) | 2% (3) | 0% (0) | 0% (1) | 0% (1) |
| Machinery, electrical equipment and metals (including transport equipment and precision instruments) | 20% (23) | 24% (27) | 29% (32) | 25% (27) | 26% (30) | 24% (26) | 22% (25) |
| Other services | 0% (1) | 0% (1) | 0% (1) | 8% (9) | 12% (14) | 17% (19) | 17% (19) |
| Textiles and leather (shoes) | 18% (21) | 10% (12) | 8% (9) | 0% (0) | 0% (0) | 0% (1) | 0% (1) |
| Transport | 3% (4) | 2% (3) | 2% (3) | 2% (3) | 2% (3) | 0% (1) | 1% (2) |
| **Total** | **100% (112)** | **100% (110)** | **100% (108)** | **100% (107)** | **100% (112)** | **100% (108)** | **100% (109)** |

*Table J: Detailed economic subsector for companies linked to at least one member of the k-shells*

| **Subsector** | **1910** | **1937** | **1957** | **1980** | **2000** | **2010** | **2015** |
| --- | --- | --- | --- | --- | --- | --- | --- |
| Food and tobacco | 6% (4) | 7% (6) | 9% (7) | 10% (7) | 2% (1) | 0% (0) | 3% (1) |
| Insurance | 11% (7) | 7% (6) | 7% (6) | 13% (9) | 13% (6) | 0% (0) | 18% (6) |
| Banks and financial companies | 26% (17) | 26% (21) | 18% (14) | 20% (14) | 23% (10) | 33% (1) | 15% (5) |
| Chemicals | 3% (2) | 5% (4) | 6% (5) | 5% (4) | 13% (6) | 0% (0) | 15% (5) |
| Construction: cement | 0% (0) | 2% (2) | 2% (2) | 2% (2) | 4% (2) | 0% (0) | 0% (0) |
| Distribution of durable and non-durable goods, food shops | 1% (1) | 0% (0) | 6% (5) | 4% (3) | 0% (0) | 0% (0) | 3% (1) |
| Energy | 4% (3) | 8% (7) | 6% (5) | 7% (5) | 4% (2) | 0% (0) | 6% (2) |
| Watchmaking | 1% (1) | 1% (1) | 2% (2) | 1% (1) | 2% (1) | 0% (0) | 0% (0) |
| Equipment: wood, paper | 1% (1) | 2% (2) | 1% (1) | 1% (1) | 0% (0) | 0% (0) | 0% (0) |
| Machinery, electrical equipment and metals (including transport equipment and precision instruments) | 23% (15) | 25% (20) | 28% (22) | 23% (16) | 20% (9) | 0% (0) | 25% (8) |
| Other services | 0% (0) | 0% (0) | 1% (1) | 4% (3) | 9% (4) | 66% (2) | 12% (4) |
| Textiles and leather (shoes) | 15% (10) | 10% (8) | 5% (4) | 0% (0) | 0% (0) | 0% (0) | 0% (0) |
| Transport | 3% (2) | 3% (3) | 3% (3) | 4% (3) | 4% (2) | 0% (0) | 0% (0) |
| **Total** | **100% (63)** | **100% (80)** | **100% (77)** | **100% (68)** | **100% (43)** | **100% (3)** | **100% (32)** |

***Online appendix 6: Details of the positions of k-shell members by sector and their multisectoriality***

*Table K: Main sector of the k-shells (details)*

| **Main Sector** | **Roles** | **1910** | **1937** | **1957** | **1980** | **2000** | **2010** | **2015** |
| --- | --- | --- | --- | --- | --- | --- | --- | --- |
| **Business (total)** |  | **80% (60)** | **87% (90)** | **54% (114)** | **51% (101)** | **75% (72)** | **47% (22)** | **80% (39)** |
|  | **Business (companies)** | **77% (58)** | **82% (84)** | **33% (69)** | **27% (53)** | **44% (42)** | **0% (0)** | **65% (32)** |
|  | CEO, chair, or board delegate (Top 110) | 40% (30) | 54% (56) | 20% (42) | 15% (30) | 24% (23) | 0% (0) | 41% (20) |
|  | Other supervisory board (Top 110) | 37% (28) | 26% (27) | 12% (26) | 7% (14) | 17% (16) | 0% (0) | 24% (12) |
|  | Other company | 0% (0) | 1% (1) | 0% (1) | 5% (9) | 3% (3) | 0% (0) | 0% (0) |
|  | ----------Supervisory board (Top 110) total | 76% (57) | 78% (80) | 28% (60) | 19% (38) | 32% (31) | 0% (0) | 49% (24) |
|  | **Business (associations)** | **3% (2)** | **6% (6)** | **21% (45)** | **24% (48)** | **31% (30)** | **47% (22)** | **14% (7)** |
|  | Top 5 | 3% (2) | 6% (6) | 18% (38) | 20% (39) | 26% (25) | 40% (19) | 14% (7) |
|  | Other business or professional association | 0% (0) | 0% (0) | 3% (7) | 5% (9) | 5% (5) | 6% (3) | 0% (0) |
| **Politics** |  | **20% (15)** | **10% (10)** | **24% (51)** | **16% (32)** | **4% (4)** | **15% (7)** | **6% (3)** |
|  | Federal government | 0% (0) | 1% (1) | 0% (1) | 1% (1) | 0% (0) | 0% (0) | 0% (0) |
|  | Cantonal government | 9% (7) | 2% (2) | 14% (30) | 11% (21) | 3% (3) | 9% (4) | 2% (1) |
|  | City government | 0% (0) | 2% (2) | 0% (0) | 0% (0) | 1% (1) | 0% (0) | 0% (0) |
|  | Party executive committee | 4% (3) | 2% (2) | 3% (7) | 2% (3) | 0% (0) | 0% (0) | 0% (0) |
|  | Cantonal government and party | 1% (1) | 2% (2) | 1% (2) | 0% (0) | 0% (0) | 0% (0) | 2% (1) |
|  | City government and party | 0% (0) | 1% (1) | 1% (2) | 0% (0) | 0% (0) | 0% (0) | 0% (0) |
|  | Only federal parliament | 4% (3) | 0% (0) | 3% (6) | 1% (2) | 0% (0) | 0% (0) | 2% (1) |
|  | Other | 1% (1) | 0% (0) | 1% (3) | 3% (5) | 0% (0) | 6% (3) | 0% (0) |
|  | ----------Federal parliament total | 17% (13) | 7% (7) | 13% (28) | 6% (12) | 0% (0) | 0% (0) | 2% (1) |
| **Unions** |  | **0% (0)** | **1% (1)** | **8% (17)** | **9% (18)** | **11% (11)** | **30% (14)** | **2% (1)** |
|  | Top 2 | 0% (0) | 1% (1) | 6% (12) | 7% (14) | 6% (6) | 21% (10) | 2% (1) |
|  | Other | 0% (0) | 0% (0) | 2% (5) | 2% (4) | 5% (5) | 9% (4) | 0% (0) |
| **Administration** |  | **0% (0)** | **0% (0)** | **8% (17)** | **19% (37)** | **5% (5)** | **6% (3)** | **0% (0)** |
|  | Central bank | 0% (0) | 0% (0) | 1% (2) | 1% (1) | 0% (0) | 0% (0) | 0% (0) |
|  | Federal department | 0% (0) | 0% (0) | 0% (0) | 2% (4) | 0% (0) | 0% (0) | 0% (0) |
|  | Federal office | 0% (0) | 0% (0) | 5% (10) | 12% (24) | 3% (3) | 4% (2) | 0% (0) |
|  | Federal department and office | 0% (0) | 0% (0) | 0% (1) | 0% (0) | 0% (0) | 0% (0) | 0% (0) |
|  | Supreme court | 0% (0) | 0% (0) | 0% (1) | 0% (0) | 0% (0) | 0% (0) | 0% (0) |
|  | Other | 0% (0) | 0% (0) | 1% (3) | 4% (8) | 2% (2) | 2% (1) | 0% (0) |
| **Academia** |  | **0% (0)** | **2% (2)** | **6% (12)** | **5% (9)** | **4% (4)** | **2% (1)** | **12% (6)** |
|  | Professor | 0% (0) | 2% (2) | 6% (12) | 4% (8) | 4% (4) | 2% (1) | 4% (2) |
|  | ETH presidency | 0% (0) | 0% (0) | 0% (0) | 0% (0) | 0% (0) | 0% (0) | 8% (4) |
|  | School of applied sciences presidency | 0% (0) | 0% (0) | 0% (0) | 1% (1) | 0% (0) | 0% (0) | 0% (0) |
|  | ----------Academic organisation total | 0% (0) | 1% (1) | 3% (7) | 1% (2) | 2% (2) | 0% (0) | 10% (5) |
| **Total** |  | **100% (75)** | **100% (103)** | **100% (211)** | **100% (197)** | **100% (96)** | **100% (47)** | **100% (49)** |

*Table L: Details of the multisectoriality of the k-shells, by sector*

| **Main Sector** | **Roles** | **1910** | **1937** | **1957** | **1980** | **2000** | **2010** | **2015** |
| --- | --- | --- | --- | --- | --- | --- | --- | --- |
| **Business** |  | **80% (60)** | **87% (90)** | **54% (114)** | **51% (101)** | **75% (72)** | **47% (22)** | **80% (39)** |
|  | **Business (companies)** | **77% (58)** | **82% (84)** | **33% (69)** | **27% (53)** | **44% (42)** | **0% (0)** | **65% (32)** |
|  | Business associations (Top 5) | 7% (5) | 13% (13) | 4% (9) | 5% (9) | 9% (9) | 0% (0) | 18% (9) |
|  | Party executive committee | 1% (1) | 1% (1) | 0% (1) | 0% (0) | 0% (0) | 0% (0) | 0% (0) |
|  | Federal parliament | 15% (11) | 6% (6) | 3% (6) | 3% (6) | 2% (2) | 0% (0) | 0% (0) |
|  | Expert committee | 11% (8) | 22% (23) | 24% (51) | 20% (40) | 9% (9) | 0% (0) | 8% (4) |
|  | Professor | 0% (0) | 0% (0) | 0% (0) | 2% (3) | 2% (2) | 0% (0) | 4% (2) |
|  | Academic organisation | 0% (0) | 0% (0) | 2% (4) | 1% (2) | 0% (0) | 0% (0) | 6% (3) |
|  | Other interest association | 4% (3) | 7% (7) | 1% (3) | 1% (1) | 13% (12) | 0% (0) | 12% (6) |
|  | **Business (associations)** | **3% (2)** | **6% (6)** | **21% (45)** | **24% (48)** | **31% (30)** | **47% (22)** | **14% (7)** |
|  | Supervisory board (Top 110) | 3% (2) | 5% (5) | 7% (14) | 6% (11) | 7% (7) | 2% (1) | 6% (3) |
|  | Party executive committee | 0% (0) | 1% (1) | 0% (0) | 1% (2) | 0% (0) | 2% (1) | 0% (0) |
|  | Federal parliament | 1% (1) | 1% (1) | 5% (10) | 4% (7) | 5% (5) | 9% (4) | 0% (0) |
|  | Expert committee | 3% (2) | 4% (4) | 20% (42) | 23% (45) | 26% (25) | 36% (17) | 8% (4) |
|  | Professor | 0% (0) | 1% (1) | 1% (2) | 0% (0) | 1% (1) | 0% (0) | 0% (0) |
|  | Academic organisation | 0% (0) | 0% (0) | 0% (1) | 1% (1) | 1% (1) | 0% (0) | 2% (1) |
|  | Other interest association | 0% (0) | 2% (2) | 1% (3) | 1% (2) | 2% (2) | 0% (0) | 0% (0) |
| **Politics** |  | **20% (15)** | **10% (10)** | **24% (51)** | **16% (32)** | **4% (4)** | **15% (7)** | **6% (3)** |
|  | CEO, chair, or board delegate (Top 110) | 3% (2) | 1% (1) | 2% (4) | 1% (1) | 0% (0) | 0% (0) | 0% (0) |
|  | Supervisory board (Top 110) | 19% (14) | 8% (8) | 13% (27) | 4% (7) | 1% (1) | 0% (0) | 2% (1) |
|  | Federal office | 0% (0) | 0% (0) | 0% (1) | 0% (0) | 0% (0) | 0% (0) | 0% (0) |
|  | Expert committee | 7% (5) | 6% (6) | 22% (47) | 15% (30) | 4% (4) | 15% (7) | 4% (2) |
|  | Professor | 0% (0) | 0% (0) | 1% (2) | 1% (2) | 0% (0) | 0% (0) | 0% (0) |
|  | Academic organisation | 1% (1) | 1% (1) | 1% (3) | 1% (2) | 0% (0) | 0% (0) | 0% (0) |
|  | Other interest association | 3% (2) | 1% (1) | 2% (4) | 2% (3) | 0% (0) | 0% (0) | 0% (0) |
| **Unions** |  | **0% (0)** | **1% (1)** | **8% (17)** | **9% (18)** | **11% (11)** | **30% (14)** | **2% (1)** |
|  | Supervisory board (Top 110) | 0% (0) | 1% (1) | 1% (3) | 3% (5) | 0% (0) | 4% (2) | 0% (0) |
|  | Federal parliament | 0% (0) | 1% (1) | 3% (6) | 1% (2) | 1% (1) | 0% (0) | 0% (0) |
|  | Expert committee | 0% (0) | 1% (1) | 8% (17) | 9% (18) | 11% (11) | 30% (14) | 2% (1) |
|  | Academic organisation | 0% (0) | 0% (0) | 0% (0) | 1% (2) | 0% (0) | 0% (0) | 0% (0) |
|  | Other interest association | 0% (0) | 0% (0) | 1% (2) | 1% (1) | 1% (1) | 0% (0) | 0% (0) |
| **Administration** |  | **0% (0)** | **0% (0)** | **8% (17)** | **19% (37)** | **5% (5)** | **6% (3)** | **0% (0)** |
|  | Supervisory board (Top 110) | 0% (0) | 0% (0) | 2% (4) | 2% (4) | 0% (0) | 0% (0) | 0% (0) |
|  | Expert committee | 0% (0) | 0% (0) | 8% (17) | 19% (37) | 5% (5) | 6% (3) | 0% (0) |
|  | Professor | 0% (0) | 0% (0) | 0% (0) | 1% (1) | 0% (0) | 0% (0) | 0% (0) |
|  | Academic organisation | 0% (0) | 0% (0) | 2% (4) | 4% (7) | 0% (0) | 0% (0) | 0% (0) |
|  | Other interest association | 0% (0) | 0% (0) | 1% (2) | 1% (1) | 0% (0) | 0% (0) | 0% (0) |
| **Academia** |  | **0% (0)** | **2% (2)** | **6% (12)** | **5% (9)** | **4% (4)** | **2% (1)** | **12% (6)** |
|  | CEO, chair, or board delegate (Top 110) | 0% (0) | 1% (1) | 0% (0) | 0% (0) | 0% (0) | 0% (0) | 0% (0) |
|  | Supervisory board (Top 110) | 0% (0) | 0% (0) | 1% (3) | 1% (2) | 1% (1) | 0% (0) | 10% (5) |
|  | Federal parliament | 0% (0) | 0% (0) | 0% (0) | 1% (1) | 0% (0) | 0% (0) | 0% (0) |
|  | Expert committee | 0% (0) | 2% (2) | 6% (12) | 5% (9) | 3% (3) | 2% (1) | 4% (2) |
|  | Other interest association | 0% (0) | 0% (0) | 1% (3) | 0% (0) | 0% (0) | 0% (0) | 2% (1) |
| **Total** |  | **100% (75)** | **100% (103)** | **100% (211)** | **100% (197)** | **100% (96)** | **100% (47)** | **100% (49)** |

***Online appendix 7: Network’s largest component***

*Figure A: Two-mode network graphs of the largest component of the Swiss elite networks*

*
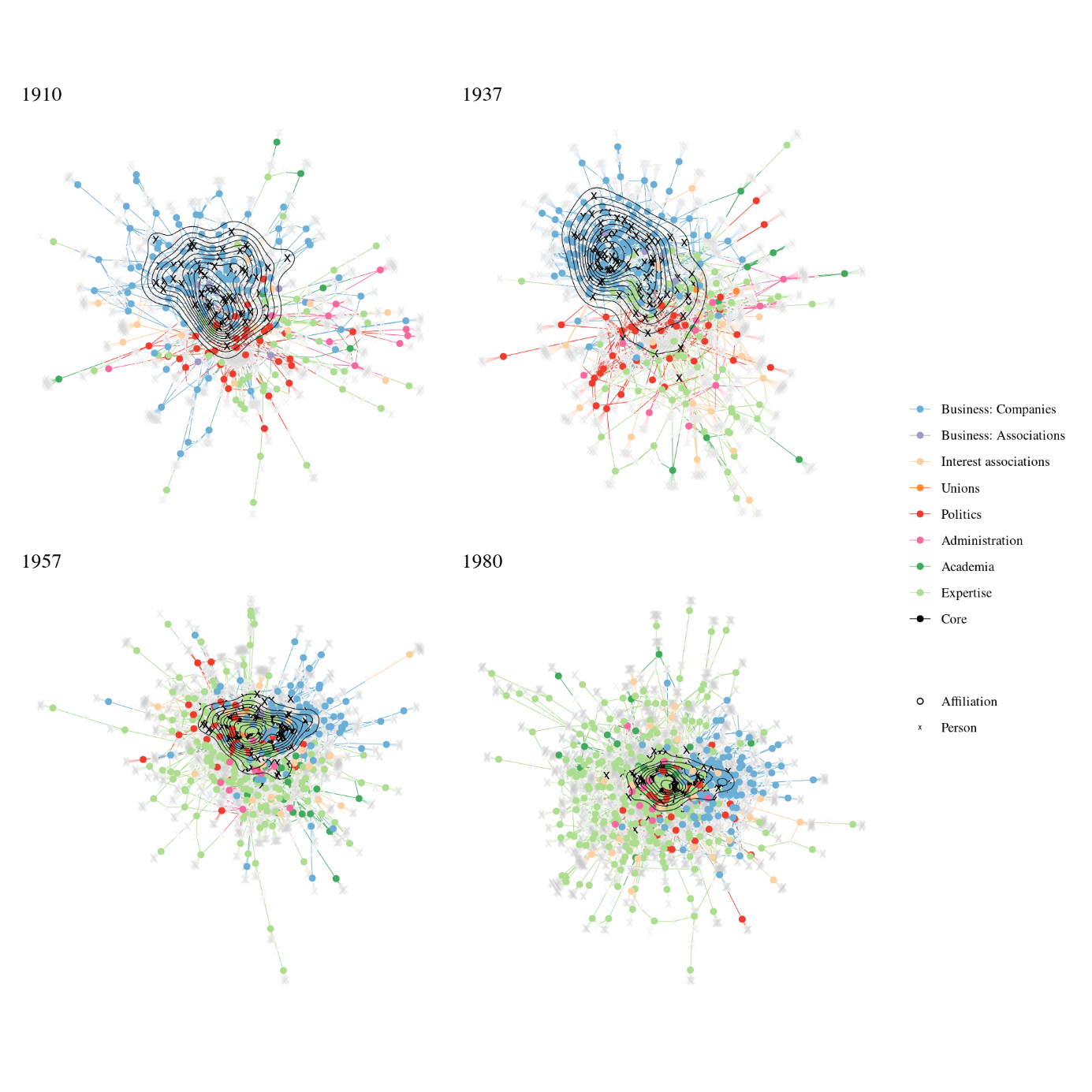
*

*Notes: The colours of the circles correspond to the sector of the affiliation and the crosses to individuals. Black crosses mark core members and grey crosses non-core individuals. Ellipses in the network mark the organisation of the core in terms of centre and periphery.*

*
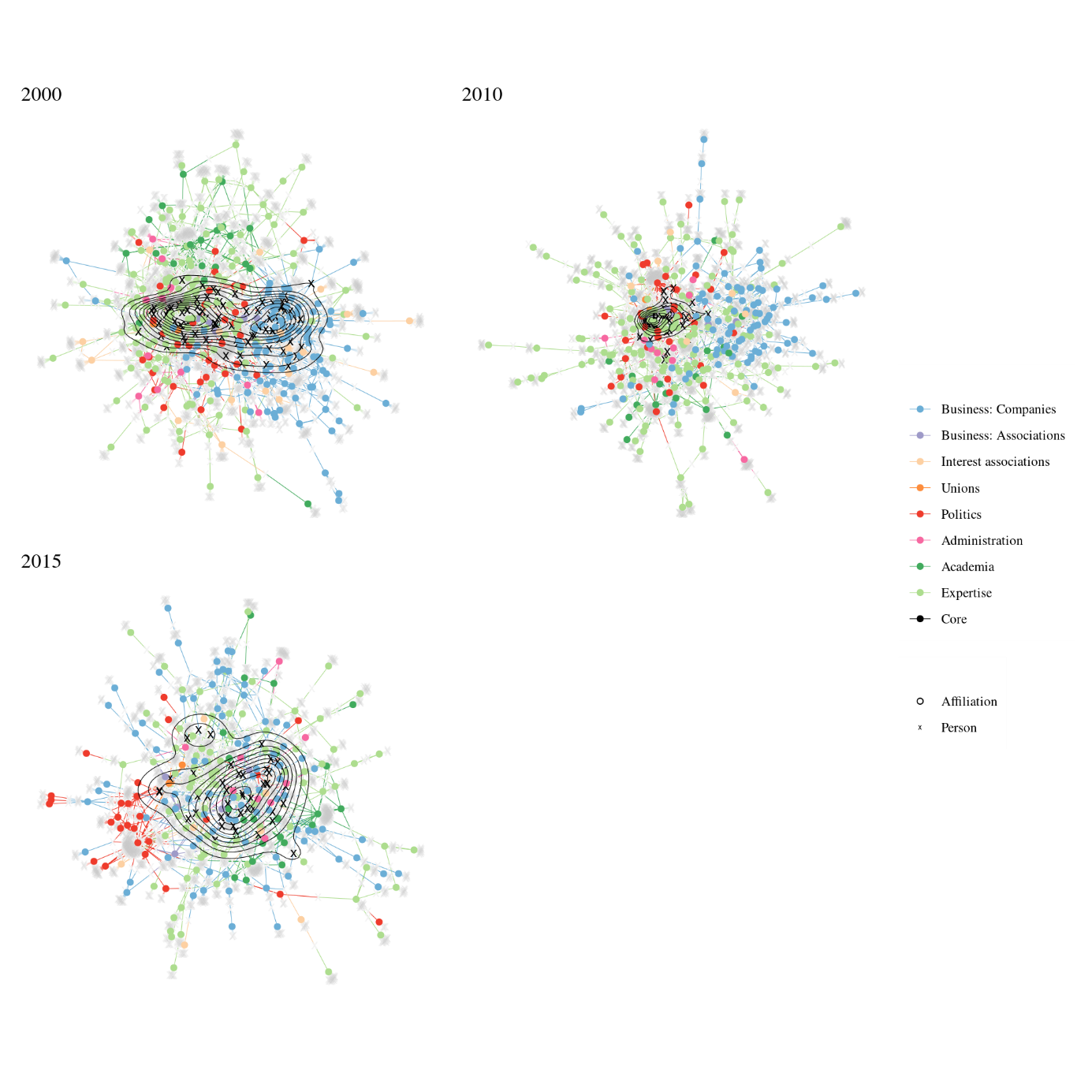
*

*Notes: The colours of the circles correspond to the sector of the affiliation and the crosses to individuals. Black crosses mark core members and grey crosses non-core individuals. Ellipses in the network mark the organisation of the core in terms of centre and periphery.*

1. S[ee: David, Thomas, André Mach, Martin Lüpold, and Gerhard Schnyder. 2015. *De La “Forteresse Des Alpes” à La Valeur Actionnariale. Histoire de La Gouvernance d’entreprise Suisse (1880-2010)*. Zurich and Geneva: Seismo; particularly the appendix pp. 473-501, for the](https://www.zotero.org/google-docs/?iAPRaL) complete methodology of the choice of the companies and their list. [↑](#footnote-ref-1)
2. Larsen, Anton Grau, and Christoph Houman Ellersgaard, “Identifying power elites—K-cores in heterogeneous affiliation networks”, *Social Networks* 50: 55-69. [↑](#footnote-ref-2)
3. Liu, Ying, Ming Tang, Tao Zhou, and Younghae Do. 2015. “Improving the Accuracy of the k-Shell Method by Removing Redundant Links: From a Perspective of Spreading Dynamics.” *Scientific Reports* 5:srep13172. doi: 10.1038/srep13172. [↑](#footnote-ref-3)
4. Garas, Antonios, Frank Schweitzer, and Shlomo Havlin. 2012. “A k -Shell Decomposition Method for Weighted Networks.” *New Journal of Physics* 14(8): 083030. doi: 10.1088/1367-2630/14/8/083030. [↑](#footnote-ref-4)
5. Malliaros, Fragkiskos D., Christos Giatsidis, Apostolos N. Papadopoulos, and Michalis Vazirgiannis. 2020. “The Core Decomposition of Networks: Theory, Algorithms and Applications.” *The VLDB Journal* 29(1):61–92. doi: 10.1007/s00778-019-00587-4; Seidman, Stephen B. 1983. “Network Structure and Minimum Degree.” *Social Networks* 5(3): 269–287. [↑](#footnote-ref-5)
